# Segmental and transmural motion of the rat myocardium estimated using quantitative ultrasound with new strategies for infarct detection

**DOI:** 10.3389/fbioe.2023.1236108

**Published:** 2023-09-07

**Authors:** Yinong Wang, Wufan Chen, Qing Wang

**Affiliations:** ^1^ School of Biomedical Engineering, Southern Medical University, Guangzhou, Guangdong, China; ^2^ Guangdong Provincial Key Laboratory of Medical Image Processing, Southern Medical University, Guangzhou, Guangdong, China; ^3^ Guangdong Province Engineering Laboratory for Medical Imaging and Diagnostic Technology, Southern Medical University, Guangzhou, Guangdong, China

**Keywords:** myocardial infarction, motion tracking, algorithm parameter optimization, transmural motion index, semiautomatic segmentation, quantitative ultrasound

## Abstract

**Introduction:** The estimation of myocardial motion abnormalities has great potential for the early diagnosis of myocardial infarction (MI). This study aims to quantitatively analyze the segmental and transmural myocardial motion in MI rats by incorporating two novel strategies of algorithm parameter optimization and transmural motion index (TMI) calculation.

**Methods:** Twenty-one rats were randomly divided into three groups (*n* = 7 per group): sham, MI, and ischemia–reperfusion (IR) groups. Ultrasound radio-frequency (RF) signals were acquired from each rat heart at 1 day and 28 days after animal model establishment; thus, a total of six datasets were represented as Sham1, Sham28, MI1, MI28, IR1, and IR28. The systolic cumulative displacement was calculated using our previously proposed vectorized normalized cross-correlation (VNCC) method. A semiautomatic regional and layer-specific myocardium segmentation framework was proposed for transmural and segmental myocardial motion estimation. Two novel strategies were proposed: the displacement-compensated cross-correlation coefficient (DCCCC) for algorithm parameter optimization and the transmural motion index (TMI) for quantitative estimation of the cross-wall transmural motion gradient.

**Results:** The results showed that an overlap value of 80% used in VNCC guaranteed a more accurate displacement calculation. Compared to the Sham1 group, the systolic myocardial motion reductions were significantly detected (*p* < 0.05) in the middle anteroseptal (M-ANT-SEP), basal anteroseptal (B-ANT-SEP), apical lateral (A-LAT), middle inferolateral (M-INF-LAT), and basal inferolateral (B-INF-LAT) walls as well as a significant TMI drop (*p* < 0.05) in the M-ANT-SEP wall in the MI1 rats; significant motion reductions (*p* < 0.05) were also detected in the B-ANT-SEP and A-LAT walls in the IR1 group. The motion improvements (*p* < 0.05) were detected in the M-INF-LAT wall in the MI28 group and the apical septal (A-SEP) wall in the IR28 group compared to the MI1 and IR1 groups, respectively.

**Discussion:** Our results show that the MI-induced reductions and reperfusion-induced recovery in systolic myocardial contractility could be successfully evaluated using our method, and most post-MI myocardial segments could recover systolic function to various extents in the remodeling phase. In conclusion, the ultrasound-based quantitative estimation framework for estimating segmental and transmural motion of the myocardium proposed in our study has great potential for non-invasive, novel, and early MI detection.

## 1 Introduction

Myocardial infarction (MI) is caused by the occlusion of coronary arteries. It is one of the most severe cardiovascular diseases and the leading cause of death worldwide. It is estimated that every 40 seconds a person is affected by MI in the United States and that approximately 14% of those with MI will eventually die as a result of MI (Tsao et al., 2023). Alternations of electrocardiography (ECG) like ST-segment elevation are commonly used for MI diagnosis (Ibanez et al., 2018). However, ECG cannot provide a definite diagnosis of ischemia. An increase in the cardiac troponin level is another biomarker for MI diagnosis, but it is not suitable for the early diagnosis of MI because the cardiac troponin level may not increase immediately after MI occurs (Konofagou et al., 2011). Coronary angiography is preferred for direct visualization of the obstructed coronary, but it is limited due to high cost and radiation exposure. The ischemic myocardium becomes passive and noncontractile even minutes after the obstruction of the coronary artery (Holmes et al., 2005; Richardson et al., 2015), indicating that the wall motion abnormality could provide a novel insight into MI diagnosis.

Myocardial elastography (ME) (Konofagou et al., 2002) is an ultrasound-based technique capable of quantitatively and noninvasively analyzing the wall motion abnormalities of the heart. The infarcted region of the myocardium can be successfully detected by ME. Reduced myocardial contractibility was found in the interventricular septum, septal wall, and anterior wall of the left anterior descending (LAD) coronary artery-ligated animal myocardial infarction model (Luo et al., 2007; Lee et al., 2011) as well as in MI patients (Lee and Konofagou, 2008). However, the interpretation of the regional motion of specific myocardium segments defined by a 16-segment model, which is recommended by the American Society of Echocardiography (ASE) and the European Association of Cardiovascular Imaging (EACI) for the regional wall motion estimation of LV (Lang et al., 2015), was either not mentioned (Luo et al., 2007) or made by subjective visualization (Lee and Konofagou, 2008; Lee et al., 2011). A segmental myocardial segmentation framework could be necessary for regional myocardial function analysis.

In addition to the segmental myocardial motion, the cross-wall motion of the myocardium is also affected by the transmural extent of MI. A systolic wall thickening gradient exists between the endocardium and epicardium, where the endocardium undergoes greater motion than the epicardium in a healthy heart (Sabbah et al., 1981; Cheng et al., 2008). Endocardial contractility is found to be affected more severely than epicardial contractility by non-transmural myocardial infarction, while both endocardial and epicardial functions are affected by transmural myocardial infarction (Maruo et al., 2007; Becker et al., 2009). In order to quantitatively measure the transmural motion gradient of the LV myocardium, the bead sets were placed at different layers of the LV myocardium as the marker for layer-specific strain calculation but with invasion (Kindberg et al., 2011). Chen and Nakatani (2011) proposed a novel index called a transmural myocardial strain gradient (TMSG) and successfully validated its ability in differentiating akinetic and normal LV segments. However, TMSG was derived from M-mode strain imaging, which is angle-dependent. A novel angle-independent quantitative biomarker for interpreting transmural myocardial motion is necessary in MI diagnosis.

Normalized cross-correlation (NCC) serves as the fundamental block matching method for motion tracking in ME, which is the most commonly used time-delay estimator, and is demonstrated to obtain the most accurate results among other motion tracking algorithms (Viola and Walker, 2003). The matching block size and overlap between successive blocks are key algorithmic parameters that determine the performance of NCC in motion tracking. A 2D matching block was found to be superior to a 1D matching block for more accurate displacement calculation, and an optimal block size was also investigated under simulated compression (Lopata et al., 2009; Li et al., 2016). The overlap between successive matching windows is negatively related to the axial resolution of the strain map obtained through calculating the gradient of displacement (Alam et al., 2000; Righetti et al., 2002; Srinivasan et al., 2003). The relationship between the overlap and the performance of NCC in displacement estimation is relatively less known from the literature. The signal-to-noise ratio (SNR) and root mean square error (RMSE) (Lopata et al., 2009; Li et al., 2016; He et al., 2017) are typically used in estimating the quality and accuracy of the calculated displacement map. However, RMSE is used in the simulation study and is not suitable for *in vivo* application due to the lack of ground truth of displacement estimation. Bayat et al. (2020) proposed a motion-compensated cross-correlation metric to estimate the reliability of uniaxial movement used in a viscoelastic model for breast cancer classification, which could be a potential metric for evaluating the accuracy of displacement calculation.

Therefore, in this study, the segmental and transmural myocardial motion was tracked based on radio-frequency (RF) data using the vectorized normalized cross-correlation (VNCC) proposed in our previous study (Wang et al., 2019). To improve its performance in the detection of MI, two novel strategies were used in this study. First, the displacement-compensated cross-correlation coefficient (DCCCC) was defined to determine the optimal value of the overlap between successive matching windows. Second, a novel angle-independent biomarker called the transmural motion index (TMI) was proposed for estimating the cross-wall transmural motion gradient. Furthermore, a framework of semiautomatic segmentation of the regional and layer-specific myocardium was presented for accurate regional wall motion analysis. Finally, our method was validated using the *in vivo* data.

## 2 Materials and methods

### 2.1 Animal care and experimental protocol

Twenty-one male Sprague–Dawley rats, weighing 213.7 ± 14.9 g, were purchased from the Laboratory Animal Center, Southern Medical University, China, and were kept in metal cages under a 12-h light–dark cycle with a standard rat diet and water *ad libitum.* These rats were randomly divided into three groups (seven rats per group): a sham group, an MI group, and an ischemia–reperfusion (IR) group. Before conducting the surgery, ethical approval was obtained from the Institutional Animal Care and Use Committee (IACUC) of Southern Medical University. Experiments on rats were performed according to the Guidelines for the Care of Laboratory Animals of the National Institutes of Health.

Rats in all groups were anesthetized with pentobarbital sodium injection (35 mg/kg intraperitoneally) (Sigma-Aldrich Corp., St. Louis, MO, United States). Hair at the rats’ chest and neck was removed using a depilatory cream. The animal’s respiration was controlled by tracheal intubation using a rodent ventilator (HX-101E, Chengdu Techman Software Co., Ltd., Sichuan, Chengdu, China). After left-sided thoracotomy, the LAD coronary artery of each rat in the MI group was permanently ligated. In the IR group, a plastic tube with the diameter of 2 mm was simultaneously ligated with the LAD coronary artery of each rat. Furthermore, after a 30-min ligation, the tube and ligation were removed for ischemia–reperfusion (Xu et al., 2014; Lindsey et al., 2021). Rats in the sham group underwent a sham operation of the LAD coronary artery without ligation. Limb-lead ECG was obtained throughout each surgery using a biological function experiment system (H-420E, Chengdu Techman Software Co., Ltd., Sichuan, Chengdu, China). ST-segment elevation was regarded as the indicator of successful ischemia. After the animal model establishment, the thoracic cavity was sewed layer by layer from ribs to skin, and the ventilator was removed. Rats were placed on a heating blanket until recovery from anesthesia. Finally, the rats were returned to metal cages with a standard rat diet and water *ad libitum* for later examination. Ultrasound scanning was scheduled for all rats at 1 day (acute myocardial infarction phase) and 28 days (remodeling phase) after the surgery (Holmes et al., 2005); a total of six groups of data are represented as Sham1, Sham28, MI1, MI28, IR1, and IR28.

### 2.2 Ultrasound examination

The Vevo2100 ultrasound imaging system (VisualSonics Inc., Canada) and the MS-250 probe (VisualSonics Inc., Canada) were used in this study for RF data acquisition. The probe has a center frequency of 21 MHz, a geometric focus of 15 mm, and a bandwidth of 13–24 MHz. ECG signals can be simultaneously monitored during the scanning.

**FIGURE 1:**
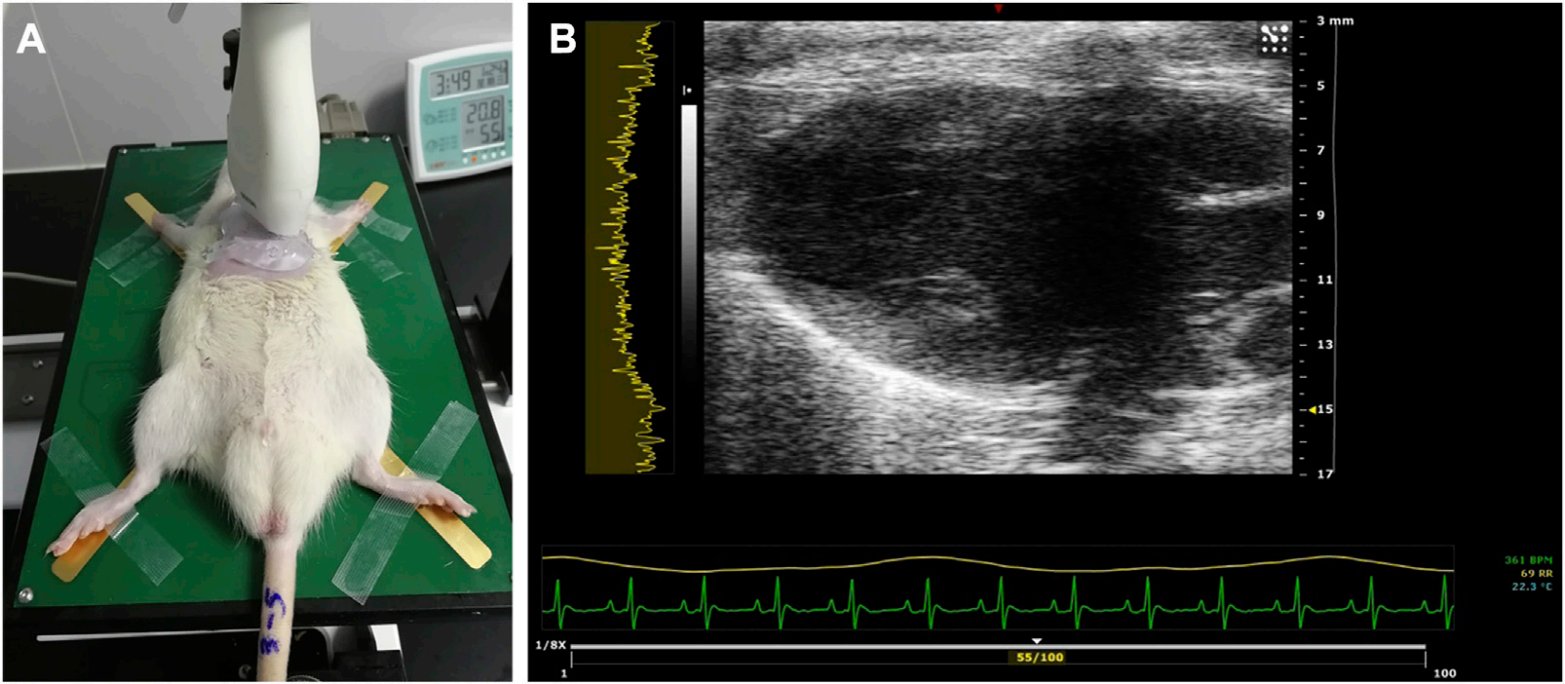
Ultrasound examination of the rat heart. **(A)** PLAX view of the left ventricle scanned using the MS-250 probe. **(B)** Typical acquired image in the PLAX view. PLAX, parasternal long axis.

All rats were first anesthetized with pentobarbital sodium injection (35 mg/kg intraperitoneally) (Solarbio Inc., Beijing, China). The limbs of the animals were fixed to the physiological monitoring platform, which is part of the imaging system, in a supine position. The parasternal long-axis (PLAX) view of the heart was scanned, and the 32-bit in-phase and quadrature (IQ) data of a total number of 100 frames of each scanning were stored and exported (Figure 1). RF data were then reconstructed from IQ data using custom software (VisualSonics Inc.).

### 2.3 Displacement estimation

The displacement of the myocardium was calculated using a VNCC-based global motion tracking algorithm proposed in our previous study (Wang et al., 2019). Assume that $F_{Pre}$ and $F_{Post}$ are two successive RF frames from the sequence of the myocardial systole period which are used for motion tracking; $F_{Pre}\left(x,y\right)$ is an RF sample value located at the xth row and yth column in $F_{Pre}$ . By using the traditional NCC-based motion tracking method, the displacement of each $F_{Pre}\left(x,y\right)$ was calculated as follows: first, a matching block (MB) centered by $F_{Pre}\left(x,y\right)$ was defined in $F_{Pre}$ , and several candidate blocks (CBs) with the same size as MB were defined in a larger region centered by current MB in $F_{Post}$ . Then, the NCC values were calculated between the current MB and each CB. Finally, the CB with the maximum NCC value was regarded as having the highest similarity to MB, and its location shifts regarding MB represent the calculated displacement results. Compared with the traditional motion tracking method using NCC, the VNCC method used here is able to obtain the NCC values for all RF samples simultaneously rather than sample by sample. Thus, this method could significantly reduce the computational time.

Due to the lower resolution in the lateral direction of RF data, we emphasized axial displacement in this study. Incremental axial displacements between each pair of successive RF frames from the systole period were calculated and then cumulated to obtain the cumulative displacement in the axial direction ( $CD_{A}$ ) which was used for segmental and transmural myocardial motion analysis.

The matching block size and overlap are key algorithmic parameters that determine the performance of VNCC in motion tracking. A matching block size of 4λ×7 pitches that was illustrated to be more appropriate in a simulated hybrid deformation scenario (Li et al., 2016) was used in this study. Here, λ is the wavelength calculated using the center frequency of the ultrasonic probe, which is 21 MHz in this study.

**FIGURE 2:**
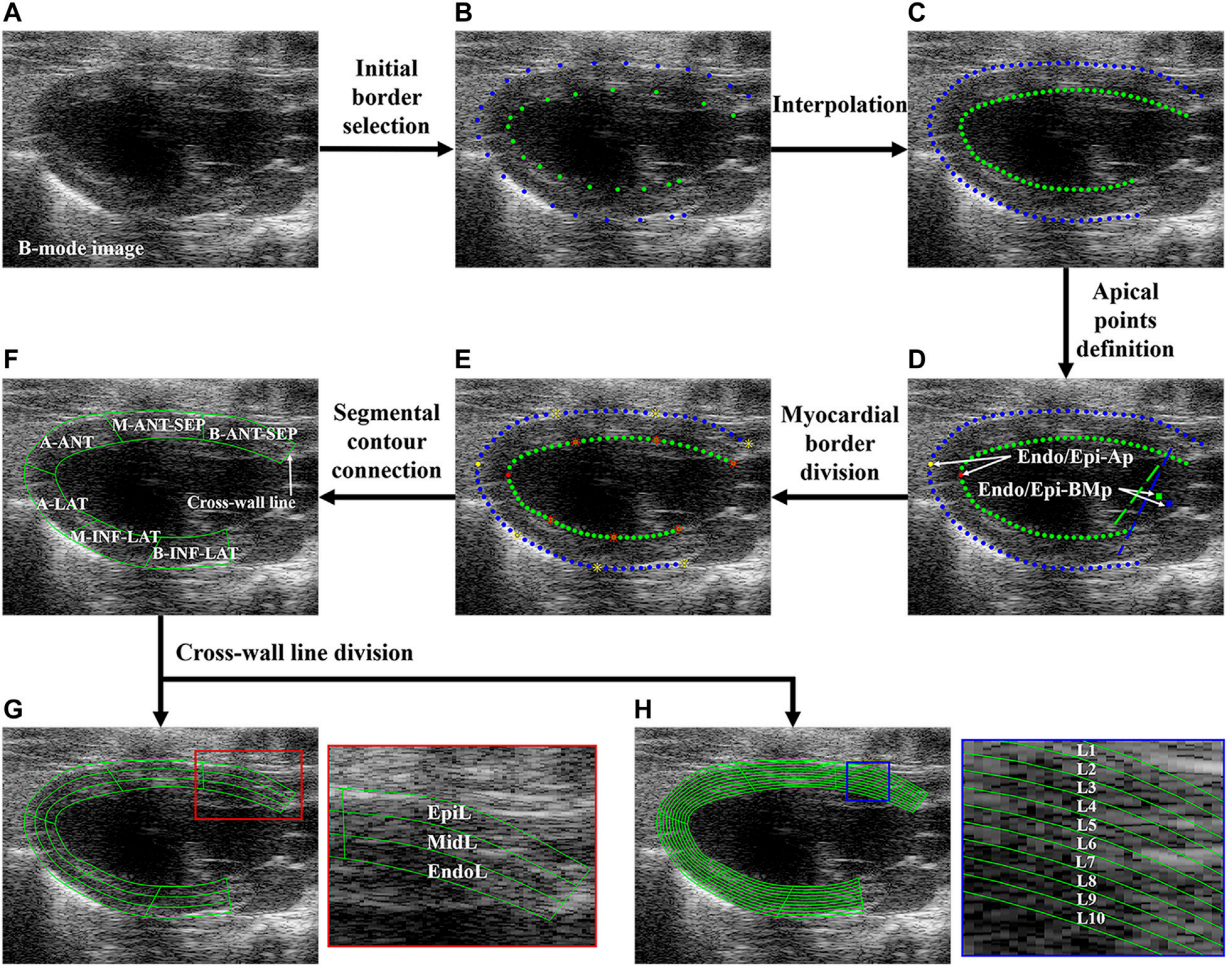
Flowchart of regional and layer-specific LV segmentation. **(A)** B-mode image; **(B)** initial borders were manually selected; **(C)** initial borders were interpolated; **(D)** Endo/Epi-Ap was defined as the farthest point from Endo/Epi-BMp; **(E)** each upper or bottom sub-borders were divided into three equal-length segments; **(F)** the borders of six LV segments were connected. Each myocardial segment was divided into three **(G)** and 10 **(H)** layers through cross-line division. LV, left ventricle; Endo/Epi-Ap, endocardial/epicardial apical point; Endo/Epi-BMp, endocardial/epicardial basal middle point; A-SEP, apical septum, M-ANT-SEP, middle anteroseptal; B-ANT-SEP, basal anteroseptal; A-LAT, apical lateral; M-INF-LAT, middle inferolateral; B-INF-LAT, basal inferolateral; EpiL, epicardial layer; MidL, middle layer; EndoL, endocardial layer; L, layer.

The values of the overlap between successive windows could be set from 10% to 99.9%. The 99.9% overlap represents that the window shift of the successive matching window is 1 RF sample. In this study, the optical value of the overlap was determined according to the quality and accuracy evaluation of the displacement calculation with different test overlap values. The quality of calculated displacement maps was evaluated using SNR. Furthermore, a displacement-compensated cross-correlation coefficient (DCCCC) was proposed for evaluating the accuracy of displacement calculation. SNR is defined as

$$SNR=\frac{D_{ROI}}{\sigma_{ROI}},$$ (1)

where $D_{ROI}$ and $\sigma_{ROI}$ are the average and standard deviation of displacement values in the region of interest (ROI), which is the myocardium region in this study, as shown in Figure 2F.

The axial ( $D_{A}$ ) and lateral ( $D_{L}$ ) displacement maps between the randomly selected successive $F_{Pre}$ and $F_{Post}$ from each group were calculated using VNCC with a test overlap value. So, the compensated $F_{Post}$ is obtained as each RF sample $F_{Post}\left(x,y\right)$ moving ${-D}_{A}\left(x,y\right)$ and ${-D}_{L}\left(x,y\right)$ in the axial and lateral directions, respectively. $F_{Post}$ with the displacement compensation can be described as $F_{PostC}$ :

$$F_{PostC}\left(x-D_{A}\left(x,y\right),y-D_{L}\left(x,y\right)\right)=F_{Post}\left(x,y\right).$$ (2)

Then, the cross-correlation between $F_{Pre}$ and $F_{PostC}$ is calculated and defined as DCCCC:

$$DCCCC=\frac{\sum_{i,j\in ROI}\left(F_{pre}\left(i,j\right)-\bar{f_{ij}}\right)\left(F_{postC}\left(i,j\right)-\bar{g_{ij}}\right)}{\sqrt{\sum_{i,j\in ROI}{\left(F_{pre}\left(i,j\right)-\bar{f_{ij}}\right)}^{2}\sum_{i,j\in ROI}{\left(F_{postC}\left(i,j\right)-\bar{g_{ij}}\right)}^{2}}},$$ (3)

where $\bar{f_{ij}}$ and $\bar{g_{ij}}$ are the mean values of RF data of ROI in $F_{pre}$ and $F_{postC}$ , respectively. The higher the DCCCC value, the more accurate the displacement estimation. In addition, the DCCCC is a ground-truth-free and angle-independent quality metric.

After the SNR and DCCCC calculations with all the overlap values for all groups were completed, the optimal value of the overlap could be selected with the compromise between the SNR and DCCCC values.

### 2.4 Regional and layer-specific myocardium segmentation

Before the segmental and transmural myocardial motion analysis, the semiautomatic regional and layer-specific segmentation of the myocardium was performed. Figure 2 shows the flowchart of the regional and layer-specific segmentation of the myocardium. Five steps of regional segmentation were performed in this study:

Step 1: Myocardial border selection and interpolation. The key points on the endocardial border (Endo-B) and epicardial border (Epi-B) of LV were manually selected on the B-mode image (Figure 2B) and then interpolated to track Endo-B and Epi-B (Figure 2C). The manual identification of myocardial borders was performed by a professional sonographer.

Step 2: Apical point definition. The basal middle points (BMps) of the endocardial border (Endo-BMp) were first defined as the middle point between the endocardial anterior basal point and posterior basal point (Figure 2D). Then, the distances between the Endo-BMp and the points of the Endo-B were calculated, and the farthest point from the Endo-BMp was defined as the apical point of the endocardial border (Endo-Ap) (Figure 2D). Similarly, the basal middle point of the epicardial border (Epi-BMp) and epicardial apical point (Epi-Ap) were determined.

Step 3: Myocardial border division. Endo-B and Epi-B were divided into two sub-borders by Endo-Ap and Epi-Ap, respectively. Then, each sub-border was further divided into three equal-length sections (Figure 2E).

Step 4: Segmental contour connection. The contours of the apical septal wall (A-SEP), middle anteroseptal wall (M-ANT-SEP), basal anteroseptal wall (B-ANT-SEP), apical lateral wall (A-LAT), middle inferolateral wall (M-INF-LAT), and basal inferolateral wall (B-INF-LAT) were obtained (Figure 2F).

Step 5: Cross-wall line division. In order to analyze the transmural systolic function of LV, the equal stratification of the myocardium was performed (Voigt et al., 2015). Each segment was further divided into three equal-width layers representing the endocardial (EndoL), middle (MidL) and epicardial (EpiL) layers of the myocardium, as shown in Figure 2G. Furthermore, each segment was divided into 10 layers for a more comprehensive analysis of transmural motion of LV (Figure 2H).

### 2.5 TMI definition

The average $CD_{A}$ in each layer (Figure 2H) was calculated as $CD_{A}\left(L_{n}\right),n=1,2,3\cdots 10$ , and a novel angle-independent quantitative biomarker called the transmural motion index (TMI) was proposed for the estimation of the cross-wall transmural motion gradient and analysis of systolic function. The TMI value of each LV segment was derived as the first derivative of the first-order polynomial interpolation of $CD_{A}\left(L_{n}\right),n=1,2,3\cdots 10$ .

MATLAB (R2021b, MathWorks Inc., Natick, MA) was used to program all the codes of displacement estimation and myocardial segmentation on a PC workstation (Intel Core i5-6500 CPU, 3.20 GHz, 12 GB RAM).

### 2.6 Histological measurements

After the ultrasound examination, the rats in all groups were euthanized with pentobarbital sodium injection (40 mg/kg intraperitoneally) (Solarbio Inc., Beijing, China) following abdominal aorta exsanguination. The hearts were excised, harvested, and fixed in 10% formaldehyde solution and then sent to the Guangzhou University of Chinese Medicine for histological measurements. Paraffin blocks were prepared for each dehydrated heart specimen. Three pieces of myocardium were cut from the apex to the base of the heart and were stained using Masson. The stained myocardium slices were observed using an optical microscope (BX63, Olympus Inc., Tokyo, Japan).

### 2.7 Statistical analysis

The Mann–Whitney *U*-test was used to investigate the statistical differences in $CD_{A}$ and TMI among different groups and ultrasound scanning time points. Inter-group comparisons were performed among the sham, MI, and IR groups from 1 day to 28 days after the surgery, respectively. Inter-time comparisons were performed among sham, MI, and IR groups from 1 day to 28 days of acquisitions. All statistical analyses were performed using SPSS (version 26, IBM, Armonk, NY, United States), and a *p-*value < 0.05 was considered a significant difference.

## 3 Results

### 3.1 Optimal overlap selection

**FIGURE 3:**
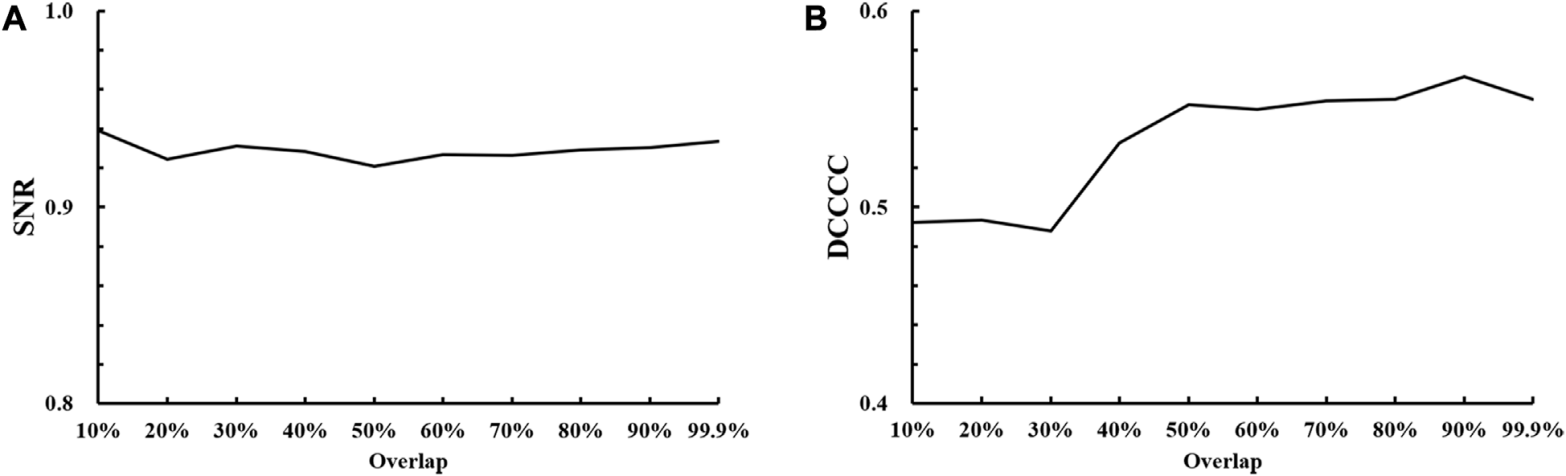
Results of optimal overlap selection with different overlap values against **(A)** SNR, signal-to-noise ratio; **(B)** DCCCC, displacement-compensated cross-correlation coefficient.

The optimal value of the overlap between successive windows was determined as the compromise between the SNR and DCCCC values. Figure 3A shows that the stable SNR values reach above 0.92 for different overlap values and are not able to provide a comparable optimal selection of the overlap. However, Figure 3B shows an obvious increase in the DCCCC value with the overlap value ranging from 30% to 50% and then a slightly stable DCCCC increase with the overlap value increasing from 50% to 80%, but a sudden increase appears at 90% and then drops immediately. So, this sudden increase might be an “outlier.” In view of a high computational cost by a higher overlap value, the optimal overlap value was 80% in this study.

### 3.2 Segmental analysis of systolic motion of LV segments

**FIGURE 4:**
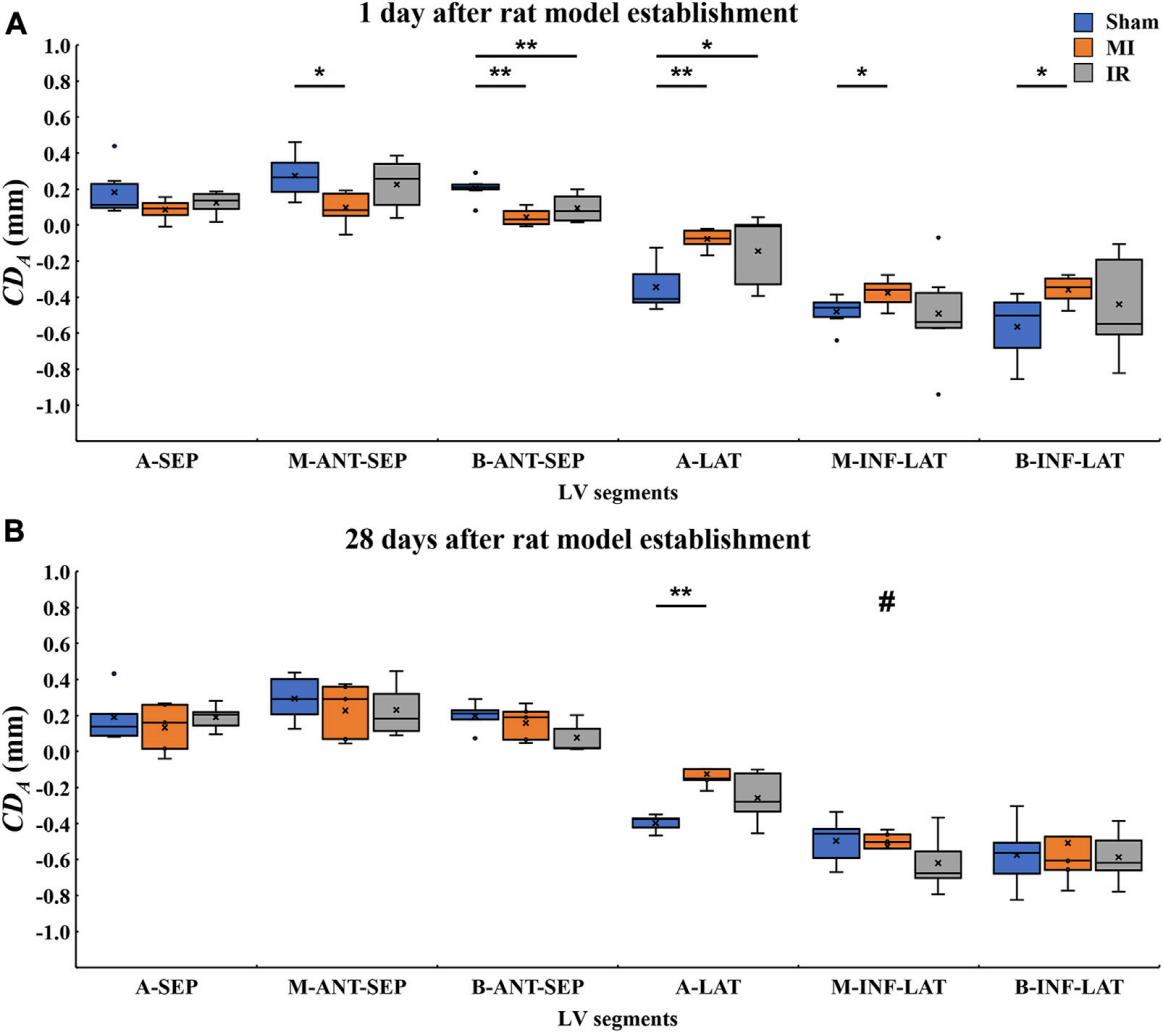
Quantitative comparisons of segmental $CD_{A}$ values among the sham, MI, and IR groups at 1 day **(A)** and 28 days **(B)** after the rat model establishment. $CD_{A},$ cumulative displacement in the axial direction; MI, myocardial infarction; IR, ischemia–reperfusion; A-LAT, apical lateral; A-SEP, apical septum; M-INF-LAT, middle inferolateral; M-ANT-SEP, middle anteroseptal; B-INF-LAT, basal inferolateral; B-ANT-SEP, basal anteroseptal. Positive and negative values on the *y*-axis represent moving downwards and upwards, respectively; × , mean displacement value of the current boxplot; #, statistically significant difference at *p* < 0.05 versus MI1; *, *p* < 0.05; **, *p* < 0.01.

In this study, we calculated the average $CD_{A}$ of each myocardial segment. Boxplots of the six segmental values from the sham, MI, and IR groups obtained between 1 day and 28 days after the surgery are shown in Figure 4, where the positive and negative $CD_{A}$ values, respectively, represent a downward and an upward movement.

#### 3.2.1 Quantitative comparisons of segmental LV systolic motion among Sham1, MI1, and IR1 groups

Figure 4A shows the 1-day acute ischemia-induced motion reduction in most myocardial segments and the recovery of motion strength due to a rapid reperfusion of blood flow. Compared with the Sham1 group, the absolute $CD_{A}$ values of the M-ANT-SEP, B-ANT-SEP, A-LAT, M-INF-LAT, and B-INF-LAT walls in the MI1 group decreased significantly (*p* = 0.011, 0.001, 0.002, 0.038, and 0.01, respectively), while the displacement value of the A-SEP segment in the MI1 group also decreased insignificantly (*p* = 0.165). The significant decrease in the absolute $CD_{A}$ value was only observed in two segments, namely, B-ANT-SEP (*p* = 0.007) and A-LAT (*p* = 0.038), between the Sham1 and IR1 groups.

#### 3.2.2 Quantitative comparisons of segmental LV systolic motion among the Sham28, MI28, and IR28 groups

After a 28-day recovery from acute ischemia, the segmental motion reductions were less obvious (Figure 4B). Compared with the Sham28 group, only the absolute $CD_{A}$ values of the A-LAT wall in the MI28 group decreased significantly (*p* = 0.008). No significant changes were found between the Sham28 and IR28 groups.

#### 3.2.3 Quantitative comparisons of segmental LV systolic motion between Sham1 and Sham28 groups, MI1 and MI28 groups, and IR1 and IR28 groups

As for inter-time comparison, no significant changes in motion of all six myocardium segments were found between Sham1 and Sham28 groups and the IR1 and IR28 groups. Compared to the MI1 group, the significant increase in the absolute $CD_{A}$ values of the M-INF-LAT (*p* = 0.03) wall and the insignificant increases in the other segments in the MI28 group suggested that the systolic function of the infarcted segments improved 28 days after MI.

### 3.3 Transmural analysis of systolic motion of LV segments

#### 3.3.1 Qualitative comparisons of transmural LV systolic motion among all groups

**FIGURE 5:**
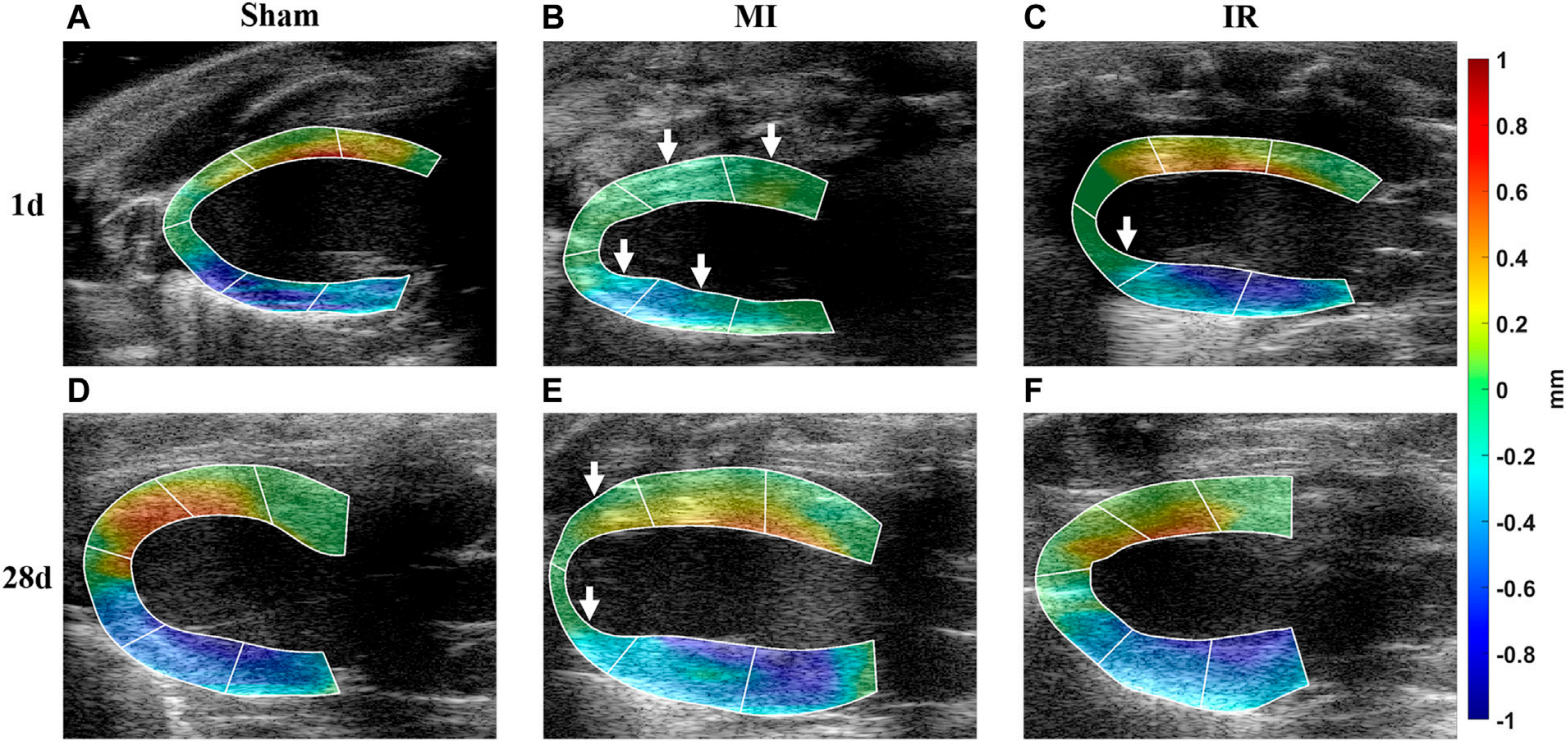
Qualitative comparisons of transmural LV systolic motion among all groups. **(A–F)** Calculated $CD_{A}$ map selected from Sham1, MI1, IR1, Sham28, MI28, and IR28 groups, respectively; $CD_{A},$ cumulative displacement in the axial direction; MI, myocardial infarction; IR, ischemia–reperfusion. Red and blue colors represent downward and upward movements, respectively. White arrows indicate the segments with obvious reduction in myocardial motion.

The calculated $CD_{A}$ maps from all groups are visualized in Figure 5. It was shown that the myocardial motion increased from the epicardial to endocardial regions in the sham group (Figures 5A, D). This transmural motion gradient was detected to be less obvious in the M-ANT-SEP, B-ANT-SEP, A-LAT, and M-INF-LAT segments in the MI1 group and the apical segments A-SEP and A-LAT in the MI28 group (Figures 5B, E) comparing to the Sham rats, whereas the transmural motion was recovered in the IR groups (Figures 5C, F). Moreover, the transmural motion strengthened in the MI28 group (Figure 5E) compared with the MI1 group (Figure 5B).

#### 3.3.2 Quantitative layer-specific comparisons of transmural LV systolic motion among Sham1, MI1, and IR1 groups

**FIGURE 6:**
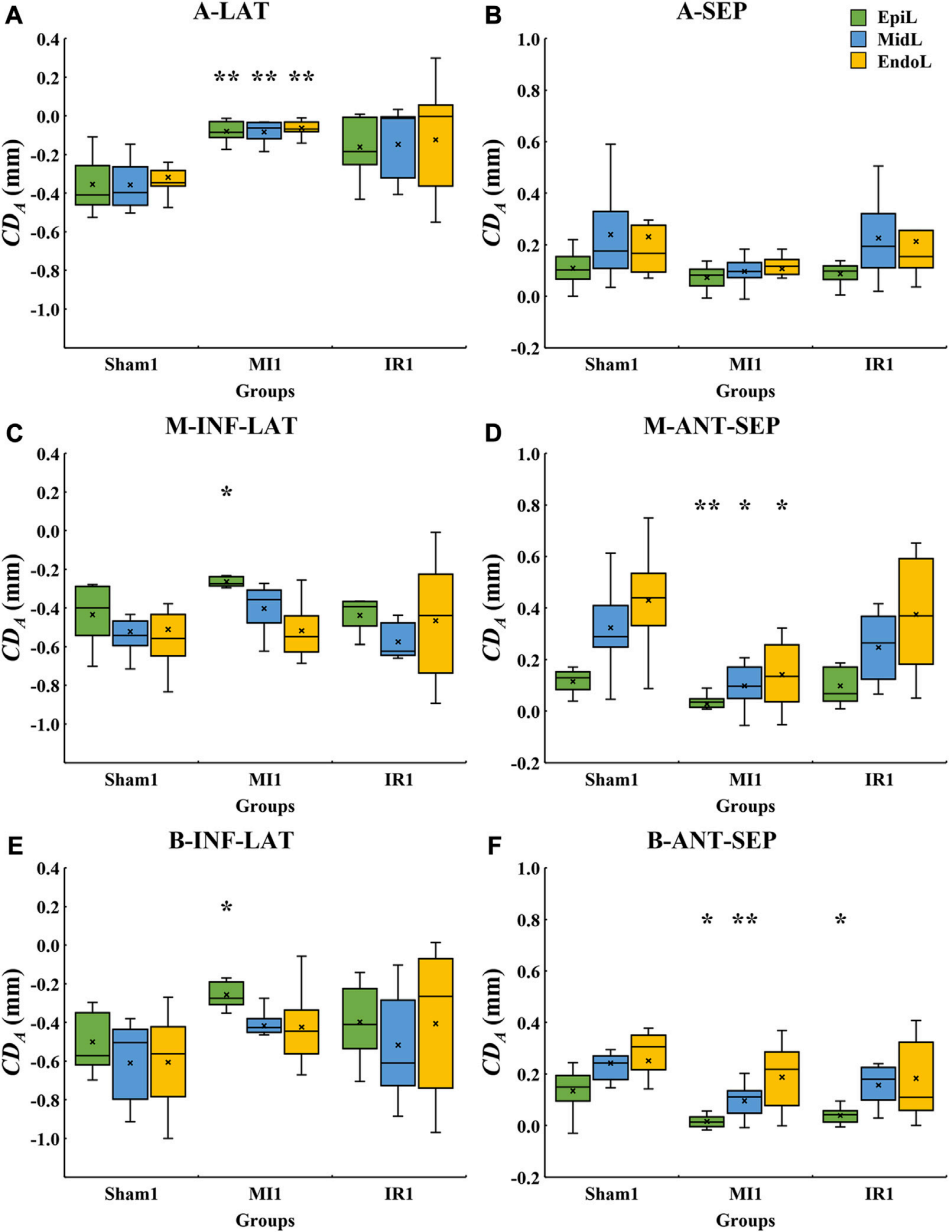
Quantitative layer-specific comparisons of transmural $CD_{A}$ values among Sham1, MI1, and IR1 groups. $CD_{A},$ cumulative displacement in the axial direction; MI, myocardial infarction; IR, ischemia–reperfusion; **(A)** A-LAT, apical lateral; **(B)** A-SEP, apical septum; **(C)** M-INF-LAT, middle inferolateral; **(D)** M-ANT-SEP, middle anteroseptal; **(E)** B-INF-LAT, basal inferolateral; **(F)** B-ANT-SEP, basal anteroseptal. Positive and negative values on the *y*-axis represent moving downwards and upwards, respectively; × , mean displacement value of the current boxplot; *, statistically significant difference at *p* < 0.05 versus Sham1; **, statistically significant difference at *p* < 0.01 versus Sham1.

The layer-specific displacements from the epicardial to endocardial layers of various LV segments from the Sham1, MI1, and IR1 groups were quantitatively compared (Figure 6). Compared with the Sham1 group, some segments in the MI1 group showed a whole transmural decrease in myocardial motion. The absolute $CD_{A}$ values of all EpiL, MidL, and EndoL of the A-LAT (*p* = 0.007, 0.002, and 0.001, respectively) and M-ANT-SEP (*p* = 0.004, 0.011, and 0.017, respectively) segments in the MI1 group (Figures 6A, D) decreased significantly. However, others showed a non-transmural decrease. The obvious motion reductions were found in the EpiL and MidL of the B-ANT-SEP wall (*p* = 0.026 and 0.007, respectively) in the MI1 group (Figure 6F). Only the EpiL of the M-INF-LAT (*p* = 0.17) and B-INF-LAT (*p* = 0.04) walls in the MI1 group (Figures 6C, F) and that of the B-ANT-SEP wall (*p* = 0.037) in the IR1 group (Figure 6F) were found decreased significantly. These results indicate that the transmural extent of infarction is more severe in the M-ANT-SEP and A-LAT walls than that in other segments.

#### 3.3.3 Quantitative layer-specific comparisons of transmural LV systolic motion among Sham28, MI28, and IR28 groups

**FIGURE 7:**
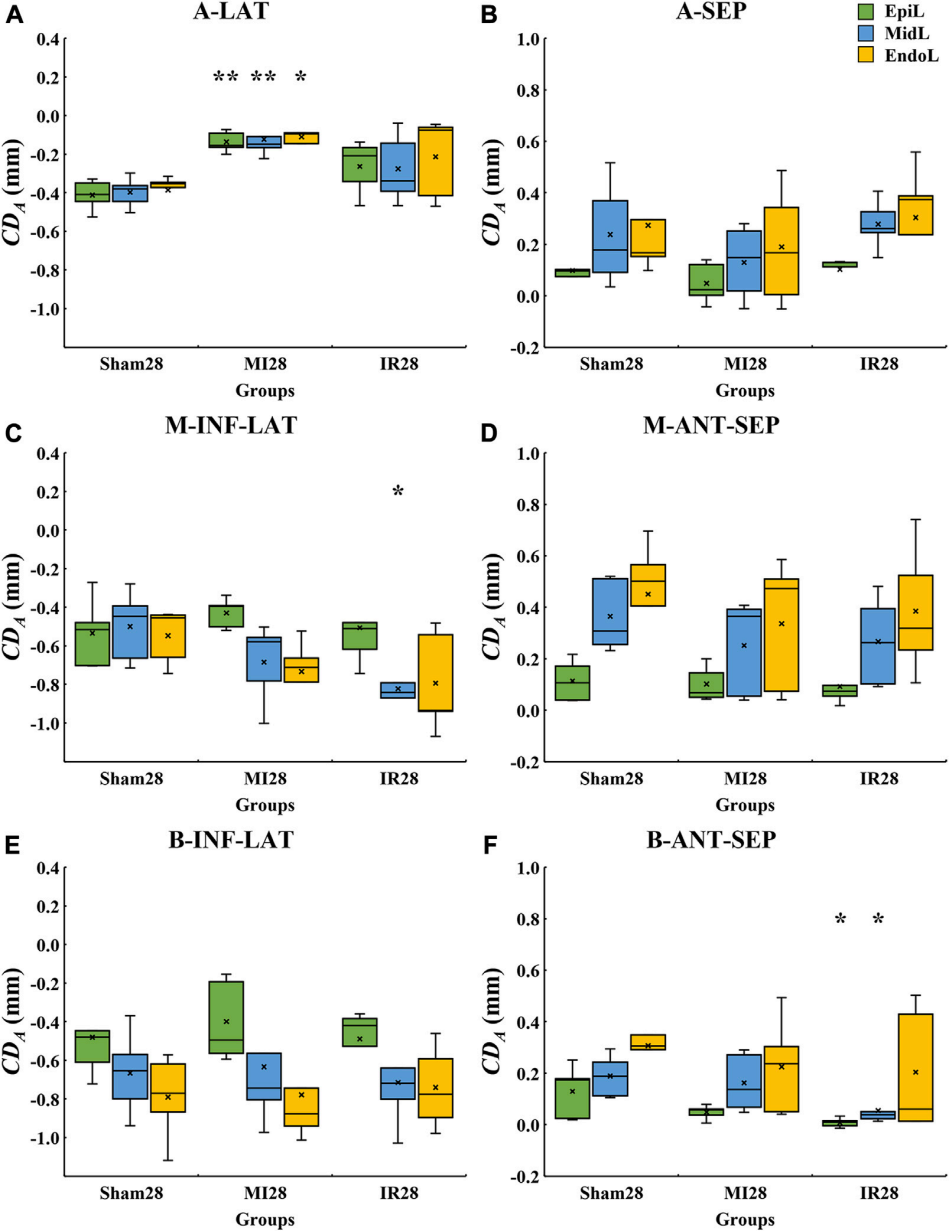
Quantitative layer-specific comparisons of transmural $CD_{A}$ values among Sham28, MI28, and IR28 groups. $CD_{A},$ cumulative displacement in the axial direction; MI, myocardial infarction; IR, ischemia–reperfusion; **(A)** A-LAT, apical lateral; **(B)** A-SEP, apical septum; **(C)** M-INF-LAT, middle inferolateral; **(D)** M-ANT-SEP, middle anteroseptal; **(E)** B-INF-LAT, basal inferolateral; **(F)** B-ANT-SEP, basal anteroseptal. Positive and negative values on the *y*-axis are the same as shown in Figure 4. × , mean displacement value of the current boxplot; *, statistically significant difference at *p* < 0.05 versus Sham28; **, statistically significant difference at *p* < 0.01 versus Sham28.

The layer-specific displacements from the epicardial to endocardial layers of various LV segments from the Sham28, MI28, and IR28 groups were quantitatively compared (Figure 7). Cross-myocardial extent of infarction was found in the A-LAT wall (Figure 7A) with the significant decrease in the absolute $CD_{A}$ values in all the three layers, EpiL (*p* = 0.008), MidL (*p* = 0.008), and EndoL (*p* = 0.016), in the MI28 group compared with the Sham28 group. For the IR28 group, less transmurality of infarction regions was noticed in the B-ANT-SEP wall with a significant reduction in $CD_{A}$ values in the EpiL (*p* = 0.038) and MidL (*p* = 0.038) compared with the Sham28 group.

#### 3.3.4 Quantitative TMI comparisons of transmural LV systolic motion among all groups

**FIGURE 8:**
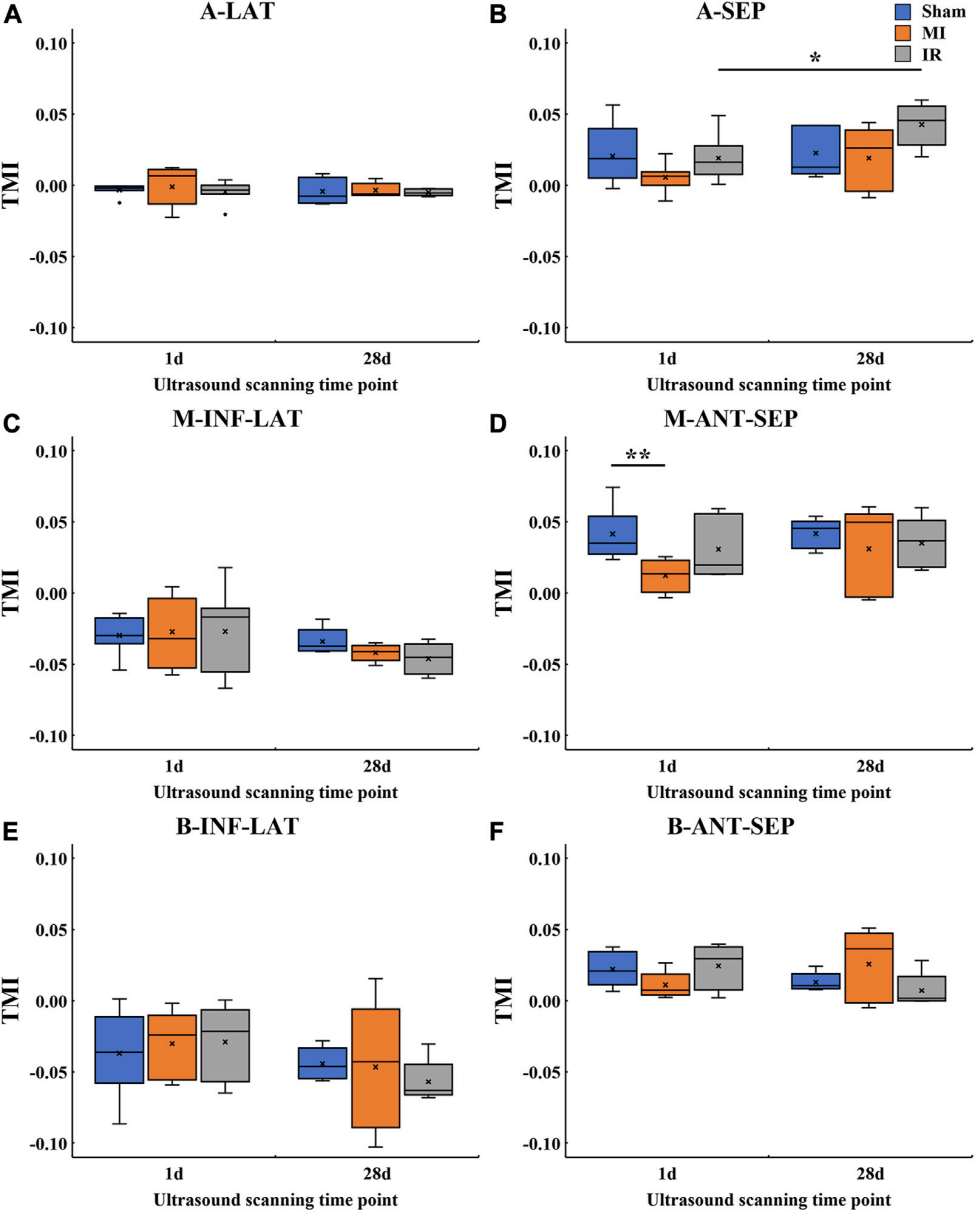
Quantitative TMI comparisons of transmural $CD_{A}$ values among all groups. $CD_{A},$ cumulative displacement in the axial direction; MI, myocardial infarction; IR, ischemia–reperfusion; **(A)** A-LAT, apical lateral; **(B)** A-SEP, apical septum; **(C)** M-INF-LAT, middle inferolateral; **(D)** M-ANT-SEP, middle anteroseptal; **(E)** B-INF-LAT, basal inferolateral; **(F)** B-ANT-SEP, basal anteroseptal; TMI, transmural motion index. × , mean TMI value of the current boxplot; *, *p* < 0.05; **, *p* < 0.01.

Furthermore, this study performed a more comprehensive analysis of transmural motion with a novel angle-independent biomarker TMI, which quantitatively described the cross-wall transmural motion gradient in all LV segments (Figure 8). No significant alterations were found in TMI of all the segments except for M-ANT-SEP. Compared with the Sham1 group, the TMI value of the M-ANT-SEP wall in the MI1 group was significantly decreased (*p* = 0.001) (Figure 8D). Furthermore, an obvious TMI increase was found from IR1 to IR28 (Figure 8B), indicating a myocardial contractility recovery from acute ischemia.

**FIGURE 9:**
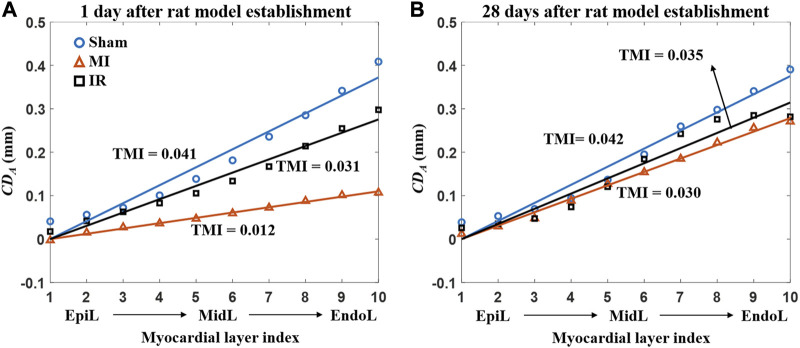
Average $CD_{A}$ plots of the 10 layers and the TMI value of the M-ANT-SEP wall for the groups at 1 day **(A)** and 28 days **(B)** after the rat model establishment. MI, myocardial infarction; IR, ischemia–reperfusion; M-ANT-SEP, middle anteroseptal; EpiL, epicardial layer; MidL, middle layer; EndoL, endocardial layer.

Figure 9 provides the detailed information of motion reduction from the epicardial to endocardial layers of the M-ANT-SEP wall. Figure 9A shows that MI induced severe attenuation in the cross-wall motion through the whole M-ANT-SEP segment (TMI = 0.012), while TMI equals to 0.041 and 0.031, respectively, in the Sham1 and IR1 groups. Figure 9B shows that the TMI values of the MI28 and IR28 groups are close to that of the Sham28 group, indicating improvement in cross-wall myocardial motion from 1 day to 28 days post-surgery.

### 3.4 Histological results

**FIGURE 10:**
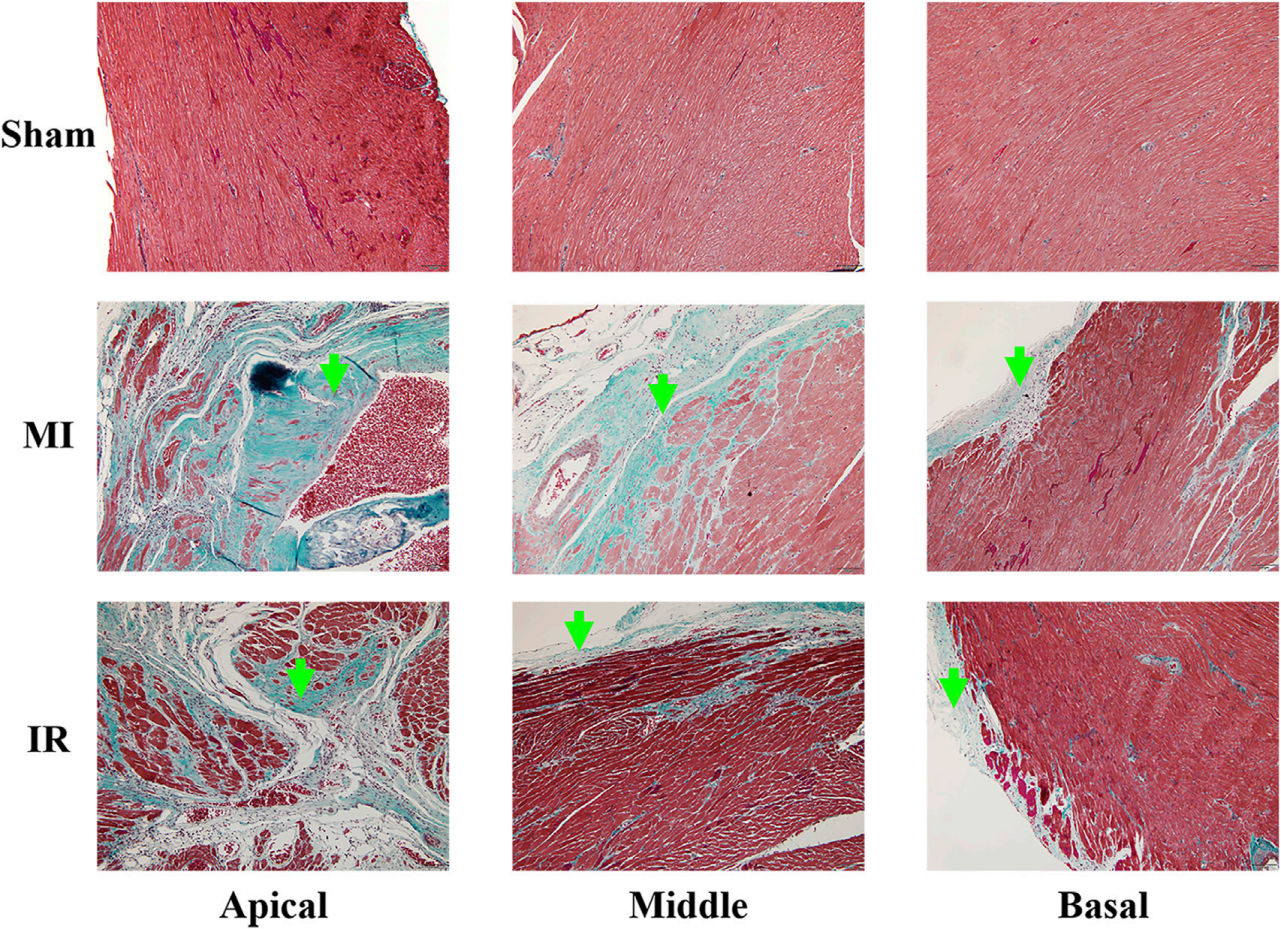
Results of Masson’s staining (×100) obtained from the apical, middle, and basal parts of the rat myocardium from the Sham, MI, and IR groups. Green arrows indicate the fibrous tissue. MI, myocardial infarction ; IR, ischemia–reperfusion.

As shown in Figure 10, the cardiomyocytes of the specimens from the sham group showed red staining and were arranged in order, indicating a healthy myocardium. Infarcted regions were found in the anterior wall and septum of the MI group, especially the apical and middle myocardium, where large regions of fibrous tissue appeared. Additionally, epicardial hyperplasia was observed in the basal myocardium of the MI group. Less severe infarction was noticed in the apex of the IR group with a relatively small region of fibrous tissue. Some epicardial hyperplasia was also observed in the middle and basal myocardia of the IR group.

## 4 Discussion

LAD coronary artery ligation is usually performed for MI animal model establishment which was used in this study. The typical perfusion territories of LAD coronary artery are A-LAT, A-SEP, M-ANT-SEP, and B-ANT-SEP walls of LV in the PLAX view of the rat’s heart (Lang et al., 2015). The results of this study show that the reduction in systolic wall motion was found in these segments of LV in the MI group compared with the sham group (Figure 4), indicating an ischemia-induced severe decrease in LV systolic function. Additionally, the M-INF-LAT and B-INF-LAT walls of LV that do not belong to the typically perfused territories of LAD coronary arteries showed significant reduction in systolic motion in the MI group. More specifically, only the epicardial layer of the M-INF-LAT and B-INF-LAT segments showed significant reduction in motion (Figures 6C, E). A previous study reported that myocardial necrosis caused by acute coronary obstruction was found to progress from the endocardium toward the epicardium (Rossello et al., 2016; Rossello et al., 2019), indicating that the contractility of the endocardium was first affected after acute coronary obstruction. This tends to conflict our findings in the M-INF-LAT and B-INF-LAT walls of LV. Due to the small size of the LAD coronary artery in the rat’s heart, commonly a larger region of the epicardium around it was ligated in the surgery to induce the obstruction of the LAD coronary artery, which could physically damage the epicardium and thus reduce its contractility. A previous study showed that the inflammation phases first occur in the progression of myocardial wound (Richardson et al., 2015). Therefore, the physical damage caused by the ligation operation or secondarily pathological damage caused by inflammation might mainly account for a more severe reduction in wall motion in the epicardial layer than in the middle and endocardial layers of the M-INF-LAT and B-INF-LAT segments.

The incremental gradient of the epicardial-to-endocardial motion decreased in the typical perfusion territories of the LAD coronary artery. Our results (Figures 6D, 8D) suggest that the transmural extent of myocardial infarction of the M-ANT-SEP wall in the MI group is more severe than that in the sham group, indicating that TMI has potential for MI diagnostics. The direction of the LV myocardial contraction is highly dependent on the orientation of myocardial fibers. The helix angle between myocardial fibers and the short-axis plane of the heart is found to have decreased from approximately +60° at the endocardium to −60° at the epicardium (Smerup et al., 2009; Toussaint et al., 2013). Therefore, the proposed TMI is a measure of radial contraction of the myocardium, which is mainly induced by the contraction of myocardial fibers from the middle-layer myocardium.

Reperfusion of the occluded LAD coronary artery benefits for post-MI myocardial contractility recovery. Significant segmental (Figure 4) and transmural (Figures 6–8) motion reductions were majorly detected in the MI group instead of the IR group, indicating the usefulness of timely reperfusion such as the percutaneous coronary intervention (PCI) (Curzen et al., 2013) procedure in the therapy of MI. A more severe myocardial motion loss in LAD coronary artery-ligated MI versus the IR rat model was also reported by Liu et al. (2020). Timely myocardial perfusion after acute myocardial ischemia could result in lower mortality (West et al., 2011).

This study focused on acute ischemia (1 day after the LAD ligation surgery) and remodeling phase (28 days after the LAD ligation surgery) (Holmes et al., 2005). The systolic contractility of LV was found restored gradually from 1 day to 28 days after the LAD ligation surgery in this study. The transmural motion reductions only occurred in fewer segments in the MI28 and IR28 groups compared with the MI1 and IR1 groups. Additionally, an improvement in segmental and transmural myocardial motions was detected in 1-day versus 28-day results. A similar increase in mechanical properties during myocardial infarction recovery was also reported in recent studies using the ME technique (Sayseng et al., 2020).

The performance of motion tracking is usually affected by parameters including systemic parameters of the imaging system, algorithmic parameters, and mechanical or acoustic parameters of the tissues (Righetti et al., 2002). Only the algorithmic parameters of NCC specific to this study, such as matching window size and overlap, could be manually selected. Li et al. (2016) conducted a systematic study on optical matching window size selection in different kinematic scenarios and found that an axial window size of 4λ (where λ is the wavelength calculated using the center frequency of the ultrasonic transducer) and a lateral window size of 7 pitches are more appropriate in the hybrid deformation scenario. In this study, we evaluated the image quality of the displacement map using SNR and the accuracy of displacement estimation using DCCCC. Compared with SNR, the newly proposed DCCCC is preliminarily more sensitive to the change in the overlap value in this study. Additionally, the DCCCC calculation does not require the ground truth of displacement, which makes it suitable for estimating the accuracy of the tracked displacements in not only simulation situations but also *in vitro* and *in vivo* scenarios. In this study, the optimal overlap value was selected as 80% in the myocardial motion tracking scenario. However, the algorithmic parameter selection is highly recommended to be explored in other motion tracking situations, such as quasi-static elastography of the thyroid and breast.

In this study, the data were acquired in the PLAX view of the heart. The myocardium in the PLAX ultrasound image is generally segmented into six segments. The histologic measurements were performed for the validation of alterations in myocardial motions by the ultrasound examination. Therefore, all 16 segments defined by the 16-segment model that is recommended by the ASE and EACI for regional wall motion estimation of LV (Lang et al., 2015) will be investigated in the future study for a more comprehensive investigation of MI-related myocardial motion evaluation.

## 5 Conclusion

In conclusion, this study evaluated the segmental and transmural motion of the LV myocardium using our VNCC motion tracking algorithm and newly proposed strategies including algorithm parameter optimization (a motion quality metric DCCCC) and transmural motion index (TMI) calculation. The results show that by using our method, the MI-induced reductions and reperfusion-induced recovery in systolic myocardial contractility could be successfully detected in the PLAX view of rat hearts. Furthermore, most post-MI myocardial segments could recover systolic function to various extents in the remodeling phase. This study suggests that the quantitative ultrasound-based motion estimation framework is feasible in investigations of the mechanical properties of the myocardium in cardiovascular diseases.

## Data Availability

The raw data supporting the conclusion of this article will be made available by the authors, without undue reservation.
